# Integrating multi-omics and machine learning methods reveals the metabolism of amino acids and derivatives-related signature in colorectal cancer

**DOI:** 10.3389/fonc.2025.1565090

**Published:** 2025-03-26

**Authors:** Jian Yue, Huiying Fang, Qian Yang, Rui Feng, Guosheng Ren

**Affiliations:** ^1^ Department of Breast and Thyroid Surgery, Chongqing Key Laboratory of Molecular Oncology and Epigenetics, The First Affiliated Hospital of Chongqing Medical University, Chongqing, China; ^2^ Department of Breast Surgery, Gaozhou People’s Hospital, Gaozhou, Guangdong, China; ^3^ Department of Breast Cancer Center, Chongqing Key Laboratory for Intelligent Oncology in Breast Cancer (iCQBC), Chongqing University Cancer Hospital, Chongqing, China; ^4^ Institute for Brain Science and Disease, Key Laboratory of Major Brain Disease and Aging Research (Ministry of Education), Chongqing Medical University, Chongqing, China

**Keywords:** metabolism, bioinformatics, RNAseq, LSM8, colorectal cancer

## Abstract

**Objective:**

The metabolism of amino acids and derivatives (MAAD) is closely related to the occurrence and development of colorectal cancer (CRC), but the specific regulatory mechanisms are not yet clear. This study aims to explore the role of MAAD in the progression of colorectal cancer and ultimately identify key molecules that may become potential therapeutic targets for CRC.

**Methods:**

This study integrates bulk transcriptome and single-cell transcriptome to analyze and identify key MAAD-related genes from multiple levels. Subsequently, numerous machine learning methods were incorporated to construct MAAD-related prognostic models, and the infiltration of immune cells, tumor heterogeneity, tumor mutation burden, and potential pathway changes under different modes were analyzed. Finally, key molecules were identified for experimental validation.

**Results:**

We successfully constructed prognostic models and Nomograms based on key MAAD-related molecules. There was a notable survival benefit observed for low-risk patients when contrasted with their high-risk counterparts. In addition, the high-risk group had a poorer response to immunotherapy and stronger tumor heterogeneity compared with the low-risk group. Further research found that by knocking down the MAAD-related gene. LSM8, the malignant characteristics of colorectal cancer cell lines were significantly alleviated, suggesting that LSM8 may become a potential therapeutic target.

**Conclusion:**

The MAAD-related gene LSM8 is likely involved in the progression of CRC and could be a hopeful target for therapeutic intervention.

## Background

CRC, as a common type of malignant tumor, poses a significant threat to human health due to its high incidence and the substantial number of deaths it causes annually. According to global cancer statistics, CRC leads to approximately one million new diagnoses each year, and its incidence is continuously increasing in many countries (1). Although early screening and surgical treatment can significantly improve patients’ survival expectations, metastasis, chemoresistance, and high recurrence rates of colorectal cancer remain the main challenges in clinical treatment (2).

The metabolism of amino acids and derivatives (MAAD) plays a crucial role in the growth, proliferation, and metastasis of tumor cells (3). Amino acids are not only the basis for protein synthesis but also promote the occurrence and progression of cancer by affecting the energy metabolism, redox balance, and immune escape mechanisms of tumor cells. Recent studies have revealed the reprogramming of amino acid metabolism in colorectal cancer cells, particularly the uptake of amino acids, metabolic pathways, and secretion of derivatives, which are of significance to tumor cells (4). Recent research has also highlighted that the reprogramming of amino acid metabolism in colorectal cancer cells, particularly the uptake, metabolic pathways, and secretion of derivatives, is crucial for the maintenance of tumor cell viability (5). The metabolism of glutamate and glutamine is vital for the energy supply and antioxidant response of colorectal cancer cells. Glutamate provides necessary energy support for cancer cells to grow rapidly by participating in the tricarboxylic acid cycle (TCA cycle) and amino acid synthesis. In addition, the metabolic products of tryptophan and tyrosine also play key roles in immune regulation and drug resistance in the tumor microenvironment (6–8). The tryptophan metabolic pathway not only promotes immune escape by regulating the function of T cells but also supports the progression of cancer by producing anti-inflammatory molecules (9).

The abnormal metabolism of amino acids not only provides the material basis for the growth of tumor cells but also affects the acid-base balance, oxygen supply, and immune response in the tumor microenvironment (10). Therefore, studying the role of amino acid metabolism in colorectal cancer, especially MAAD, has become an emerging direction in cancer treatment. The progress in multi-omics fields has been exceedingly rapid, and the combination of genomics, transcriptomics, and metabolomics provides new opportunities to reveal potential targets in these metabolic pathways (11, 12). In addition, machine learning and artificial intelligence methods can also help identify characteristic metabolic patterns in colorectal cancer patients and promote the realization of personalized treatment (13). However, the role of MAAD in the clinical prognosis and treatment response of colorectal cancer remains unclear.

To further explore the potential of MAAD as a biomarker and therapeutic target, we conducted multi-omics analyses to comprehensively investigate the significance of MAAD in CRC patients. Firstly, based on the changes in gene expression at the transcriptome level, we identified MAAD genes associated with the CRC process. Subsequently, we employed various machine learning methods to successfully construct a reliable MAAD-related prognostic model and evaluated its clinical value in CRC patients, facilitating clinical application through the construction of nomograms. Finally, we analyzed the relationships between tumor heterogeneity, immune therapy response, immune microenvironment, and single nucleotide variants (SNVs) in the high and low-risk groups of the prognostic model. Cellular experiments verified the impact of the MAAD-related gene LSM8 on cell line.

## Methods

### Data collection and processing

The TCGA-COAD(colon adenocarcinoma)was filtered to exclude patients who had received radiotherapy or chemotherapy, as well as those with missing OS data, resulting in a final cohort of 420 samples for analysis. The IMvigor210 cohort was utilized to assess the efficacy of immune therapy responses. From the GEO database, we downloaded GSE17537 (comprising 55 samples) and single-cell data from CRC patients (GSE231559, including GSM7290763, GSM7290769, GSM7290772, GSM7290773, GSM7290774, GSM7290777). In the analysis of single-cell transcriptomic data, we initially performed quality control (QC). The specific steps included filtering out cells with mitochondrial gene content exceeding 250 and ensuring that each gene was detected in at least three cells. Subsequently, we used the Seurat package to identify highly variable genes, focusing on the top 2000 most variable genes for subsequent analyses. The “AddModuleScore” function of Seurat was used to annotate the activity of the MAAD gene set (**Supplementary Table S1**). Genes that were differentially expressed between cells with high and low scores in amino acid and derivative metabolism were inferred to be key regulators of this metabolic process and were further incorporated into WGCNA whole-transcriptome analysis and pathway enrichment analysis.

### Weighted gene co-expression network analysis

WGCNA was employed to identify core modules closely associated with amino acid and derivative metabolism (14). By leveraging the modular framework of gene co-expression, we focused on biologically relevant gene clusters rather than isolated individual genes, incorporating them into subsequent analyses to enhance the credibility of our research conclusions.

### Construction of MAAD prognostic model based on machine learning

Differential analysis of the TCGA-COAD dataset identified genes with |logFC| > 0.5 and p-values < 0.05 as differentially expressed genes, which were then intersected with genes from modules related to amino acid metabolism. These intersecting genes are potentially critical in tumor development. The TCGA-COAD dataset was randomly divided into a training set (2/3) and a validation set (1/3), with the GSE17537 dataset used for external validation. Ten machine learning methods were applied, and models were trained and optimized using ten-fold cross-validation. The performance of each model across datasets was assessed using the C-index to select the best predictive model.

### Genomic variation analysis under MAAD mode

There is significant individual variation among cancer patients, and this heterogeneity poses a major challenge to tumor treatment. After comparing the DNA of cancer-adjacent and tumor samples, we calculated the Mutant Allele Tumor Heterogeneity (MATH) score and drew survival curves based on the MATH score (15). We performed copy number variation (CNV) analysis on the 12 genes with the greatest differences between the high and low-risk groups. Additionally, we used the maftools package to draw the mutation landscape of the high and low-risk groups.

### Construction of nomogram and analysis of immune cell infiltration

The survival curves for each group were plotted using the R package survminer, and the impact of MAAD on clinical characteristics was investigated. After integrating decision curve analysis (DCA), calibration curves, and C-index analysis, a nomogram was constructed to provide auxiliary clinical diagnosis for colorectal cancer patients. Additionally, the differences in immune cell infiltration between high and low-risk groups under the MMAD mode were assessed using the CIBERSORT algorithm and ssGSEA algorithm (16).

### CCK-8 and colony formation assays

Human CRC cell lines were purchased from ATCC. To measure cell viability, samples in 96-well plates (1.5 × 10^3^ cells/well) were tested at four time points: 0, 24, 48, and 72 hours. For each well, 100 μL of fresh medium containing 10 μL of CCK-8 reagent was added, and the plates were incubated at 37°C in the dark for 2 hours. The absorbance at 450 nm was measured using a microplate reader (Thermo Fisher Scientific, USA). For the colony formation assay, cells were seeded in 6-well plates at a density of 1.5 × 10^3^ cells/well. After 2 weeks, the cells were stained with crystal violet (Beyotime, China).

### Transwell assay and EdU assay

Cellular migratory capacity was evaluated utilizing Transwell inserts (8 μm pores, Labselect, China). The invasive potential was determined using inserts pre-coated with a basement membrane matrix. A volume of 100 μL of serum-deprived cell suspension, containing 2 × 10^4^ RKO cells, was placed into the upper chamber, whereas 500 μL of medium supplemented with 20% fetal bovine serum was added to the lower chamber. Following incubation (24 hours for RKO cells), cells were fixed and stained. For quantification, the mean cell counts from five randomly chosen fields with consistent cell distribution was determined. In the EdU incorporation assay, cells were incubated with EdU (Beyotime, China) at 37°C for 2 hours, subsequently fixed, permeabilized, and processed with a click chemistry reaction buffer and DAPI.

### Construction of LSM8 knockdown RKO cell lines and qRT-PCR validation

To knock down the LSM8 gene in the RKO cell line, small interfering RNA (siRNA) was utilized with the following primer sequences: Si1: TACATCAGATGGGAGAATGATTG, Si2: AGGTTTTGACCAGACCATTAATT. Transfection of RKO cells was carried out using Lipofectamine 3000 (Invitrogen, USA). The expression of LSM8 was analyzed by RT-qPCR. RNA was extracted using TRIzol reagent (Invitrogen, USA). The RNA samples were reverse transcribed into cDNA using a reverse transcription kit from Promega. For qRT-PCR analysis, SYBR Green (Abclonal, China) was mixed with cDNA in the reaction mixture. The analysis was performed using a real-time fluorescence quantitative PCR instrument (Thermo Fisher Scientific, USA) with the following LSM8 qPCR primer sequences: Forward primer F: GCCCTACTCGTTGTGGTTCA, Reverse primer R: AGAGACTCATCCCAGCAGGT.

### Wound-healing assay

RKO cells were plated in six-well culture plates and cultured until they formed a confluent monolayer. Subsequently, a scratch was made in the cell layer using a 200 μL sterile pipette tip under aseptic conditions. The cells were then cultured in 1640 medium supplemented with 2% serum. After 24 hours, images of randomly selected areas from each group were captured.

### Statistical analysis

Data analysis was performed employing GraphPad Prism 7 and R software, version 4.1.2. To discern significant quantitative disparities in variables exhibiting normal distribution, we implemented a two-tailed t-test or a one-way ANOVA. Conversely, for variables with non-normal distribution, we opted for the Wilcoxon test or the Kruskal-Wallis test as appropriate. The threshold for statistical significance was set at p < 0.05, denoted by * for p < 0.05, ** for p < 0.01, *** for p < 0.001, and **** for p < 0.0001, with ns indicating no statistical significance.

## Results

### Characteristics of MAAD in colorectal cancer single-cell sequencing samples

**Figure 1:**
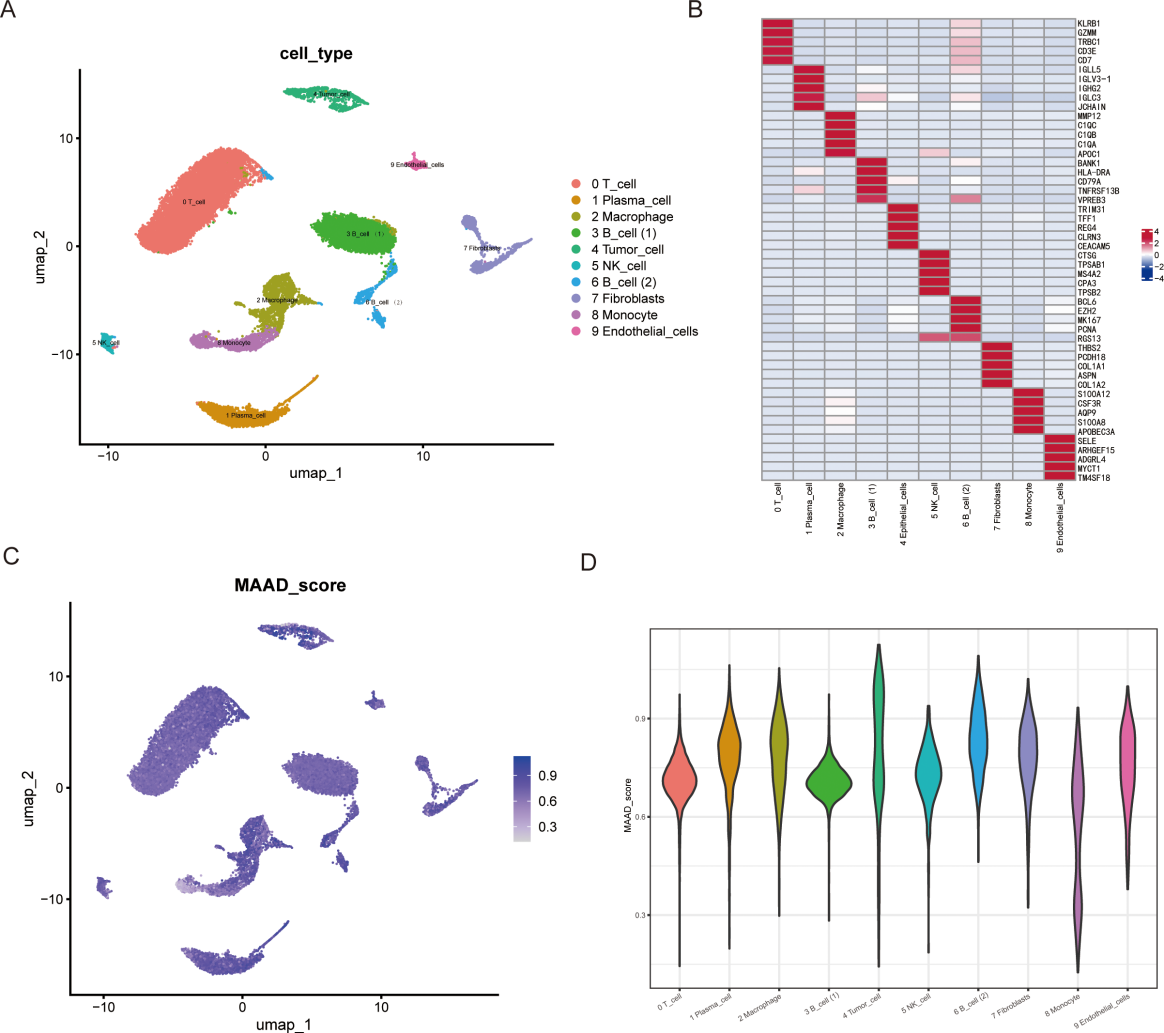
UMAP visualization and MAAD score of various cell types. **(A)** UMAP projection displaying the clustering of various cell types in two-dimensional space. Each point represents a single cell, and cells are color-coded based on their type. The cell types include T cells, plasma cells, macrophages, two distinct B cell subpopulations, tumor cells, NK cells, fibroblasts, monocytes, and endothelial cells. **(B)** Heatmap overlay of the gene expression score on the UMAP plot. The color gradient indicates the relative expression level of key genes across the cell types. **(C)** Quantitative representation of the MAAD score distribution across different cell types, with the average gene expression score shown for each cell type. **(D)** The violin plot illustrates the MAAD score of different cell types.

To investigate the role of MAAD in colorectal cancer, we performed dimensionality reduction on single-cell data from colorectal cancer using PCA and UMAP, thereby reducing noise and redundancy in the data while preserving local similarities between data points. Subsequently, we identified T cells, Plasma cells, Macrophages, B cells (1), B cells (2), Tumor cells, NK cells, Fibroblasts, Monocytes, and Endothelial cells in the immune microenvironment of colorectal cancer patients (**Figure 1A**). The heatmap in **Figure 1B** displays the top 5 marker genes for each cell subpopulation. We then scored all cell subpopulations against the 408 genes in the MAAD gene set (**Figure 1C**). The violin plot (**Figure 1D**) shows that B cells (2), Macrophages, and Endothelial cells exhibit higher levels of MAAD activity. Based on the MAAD activity scores, we divided all cells into high-MAAD and low-MAAD groups. The differentially expressed genes between the high-MAAD and low-MAAD groups are considered to potentially influence the MAAD process.

### Identification of key MAAD-related genes

**Figure 2:**
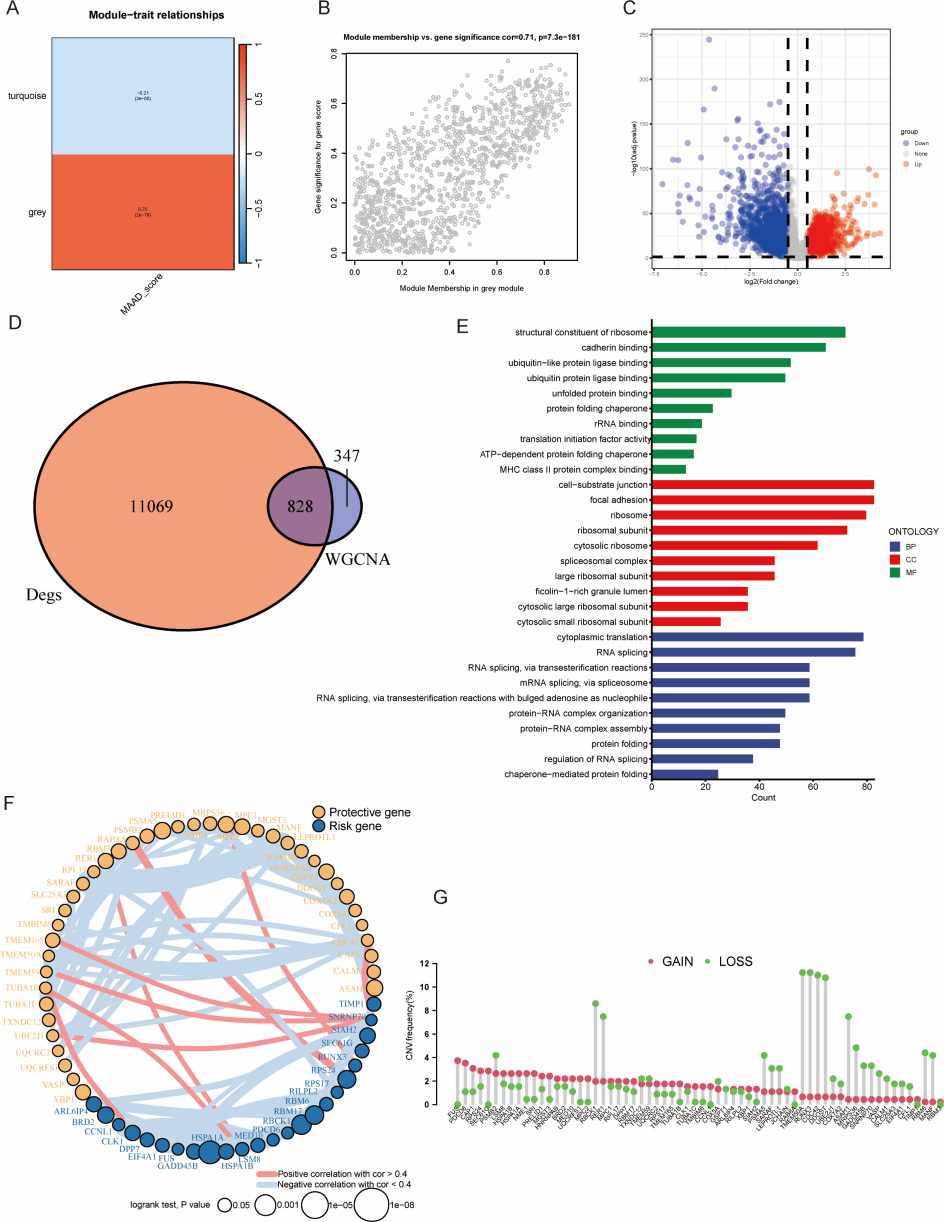
WGCNA analysis and functional annotation of gene modules. **(A)** WGCNA shows the correlations between gene co-expression modules and specific phenotypes. Red indicating positive correlations and blue indicating negative correlations. **(B)** Scatter plot showing the relationship between module membership and gene significance for the grey module. The x-axis represents the module membership score, while the y-axis represents the gene significance. **(C)** Volcano plot of differential gene expression analysis (DEG). The x-axis shows the log2 fold change, while the y-axis shows the statistical significance (–log10 adjusted p-value). **(D)** The Venn diagram shows the overlap between WGCNA module genes and differentially expressed genes. **(E)** Gene ontology (GO) enrichment analysis was performed for the functional categories of the overlapping genes. **(F)** The results of univariate Cox regression analysis for MAADRG and their correlation analysis. **(G)** CNV frequency of MAADRG.

To better understand the role of in the progression of colorectal cancer, we utilized the ssGSEA algorithm to score MAAD in TCGA-COAD for subsequent analysis. Following WGCNA analysis, we identified two modules related to MAAD (**Figure 2A**). The module membership within the grey module was highly positively correlated with the Gene significance MAAD score (**Figure 2B**; detailed information for the grey module is provided in **Supplementary Table S2**). Subsequently, we conducted an analysis of gene expression to pinpoint genes that exhibit varied expression levels within the TCGA-COAD dataset (**Figure 2C** and detail in **Supplementary Table S3**). By intersecting these differentially expressed genes with the WGCNA grey module, we obtained 828 genes (**Figure 2D**), which we defined as MAAD-related genes (MAADRG). Gene ontology analysis revealed that these genes were involved in biological processes (BP) such as structural constituent of ribosome, cellular components (CC) like cell−substrate junction, and molecular functions (MF) including RNA splicing, via transesterification reactions (**Figure 2E**). Through univariate Cox regression analysis, we narrowed down the 823 MAADRG to 64 (**Figure 2F**, which illustrates the relationships among these 64 genes). Upon conducting CNV frequency analysis on these 64 genes, we observed an interesting phenomenon: the copy number of TEME50A, RUNX3, CDC42, and DDOST all decreased by approximately 10% (**Figure 2G**).

### Construction and validation of prognostic models based on multiple machine learning

**Figure 3:**
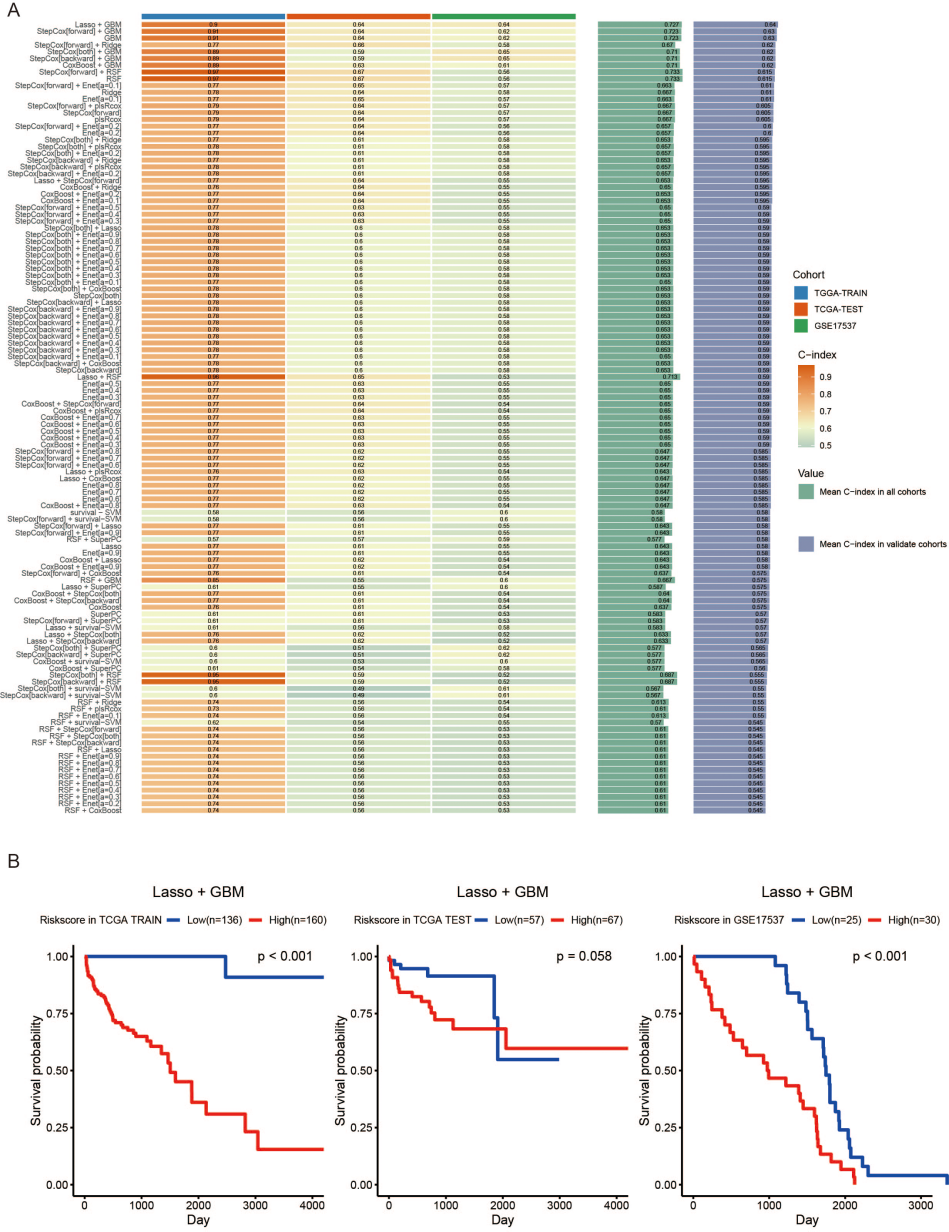
Construction of a MAAD prognostic model based on machine learning. **(A)** MAAD prognostic model 101 prognostic models were developed and C-index scores were assigned to the different models. **(B)** OS Kaplan–Meier survival curves in TCGA and GEO datasets based on the MAAD prognostic model.

**Figure 4:**
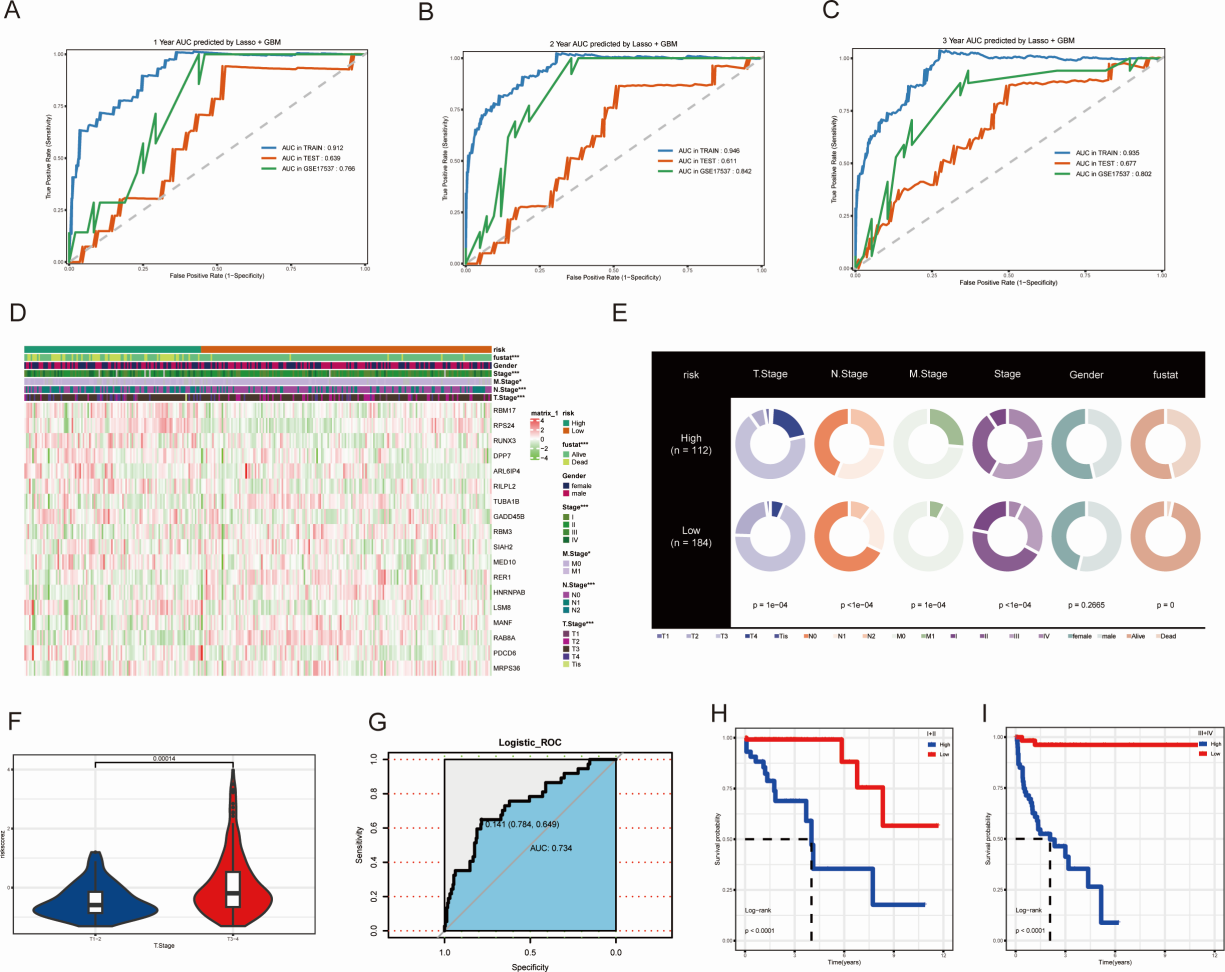
Validation of the MAAD prognostic model across multiple datasets. **(A-C)** ROC curves for 1-3 years OS predictions of CRC patients under the MAAD prognostic model. **(D)** Heatmap showing the expression levels of key genes included in the MAAD model across the high-risk, low-risk groups and clinical variables. **(E)** Distribution of clinical features across risk groups. **(F)** Risk scores for T1-2 and T3-4. **(G)** MAAD prognostic model ROC curves for predicting T stage. **(H, I)** Kaplan-Meier analysis of the MAAD prognostic model in colorectal cancer patients stratified by T stage. *P<0.05, ***P<0.001.

We analyzed the previously filtered 64 genes using 101 machine learning methods for subsequent analysis. The TCGA-COAD dataset was randomly divided into a training set and an internal validation set at a ratio of 2:1. Additionally, GSE17537 was used as an external dataset to further validate the reliability of the models. Subsequently, the C-index was calculated for both the training and validation sets of the 101 prognostic models (**Figure 3A**). We found that the Lasso + GBM, StepCox [forward] + GBM, GBM, and CoxBoost + GBM models had C-index values greater than 0.6 in the TCGA-TRAIN, TCGA-TEST, GSE17537, Mean C-index in all cohorts, and Mean C-index in validate cohorts (**Figure 3A**). The Lasso + GBM model had the highest C-index score, so we used this model for the MAAD prognostic model. We found that patients with low MAAD risk scores had significantly better overall survival (OS) compared to those with high risk scores in the TCGA TRAIN and GSE17537 cohorts, with P = 0.058 in the TCGA TEST cohort (**Figure 3B**). ROC analysis results showed that our MAAD prognostic model had good predictive power in the TCGA TRAIN, TCGA TEST, and GSE17537 cohorts (**Figures 4A-C**). Changes in clinical indicators are of significant guiding importance for the treatment of cancer patients (**Figures 4D, E**). Therefore, we analyzed the changes in T, N, M staging, stage, and gender between the high and low MAAD risk groups. Apart from the gender indicator, the rest of the indicators revealed substantial variance when comparing the high and low risk groups. We were pleased to find that patients with T3-T4 had higher risk scores compared to those with T1-T2 (**Figure 4F**). Additionally, the MAAD prognostic model can be used to predict the T stage of colorectal cancer patients, and survival analysis results also showed that the MAAD prognostic model had good predictive performance for T1-T2 and T3-T4 colorectal cancer patients (**Figures 4G-I**). These results indirectly prove the reliability and applicability of our model.

### Establishment and validation of a MAAD-related nomogram

**Figure 5:**
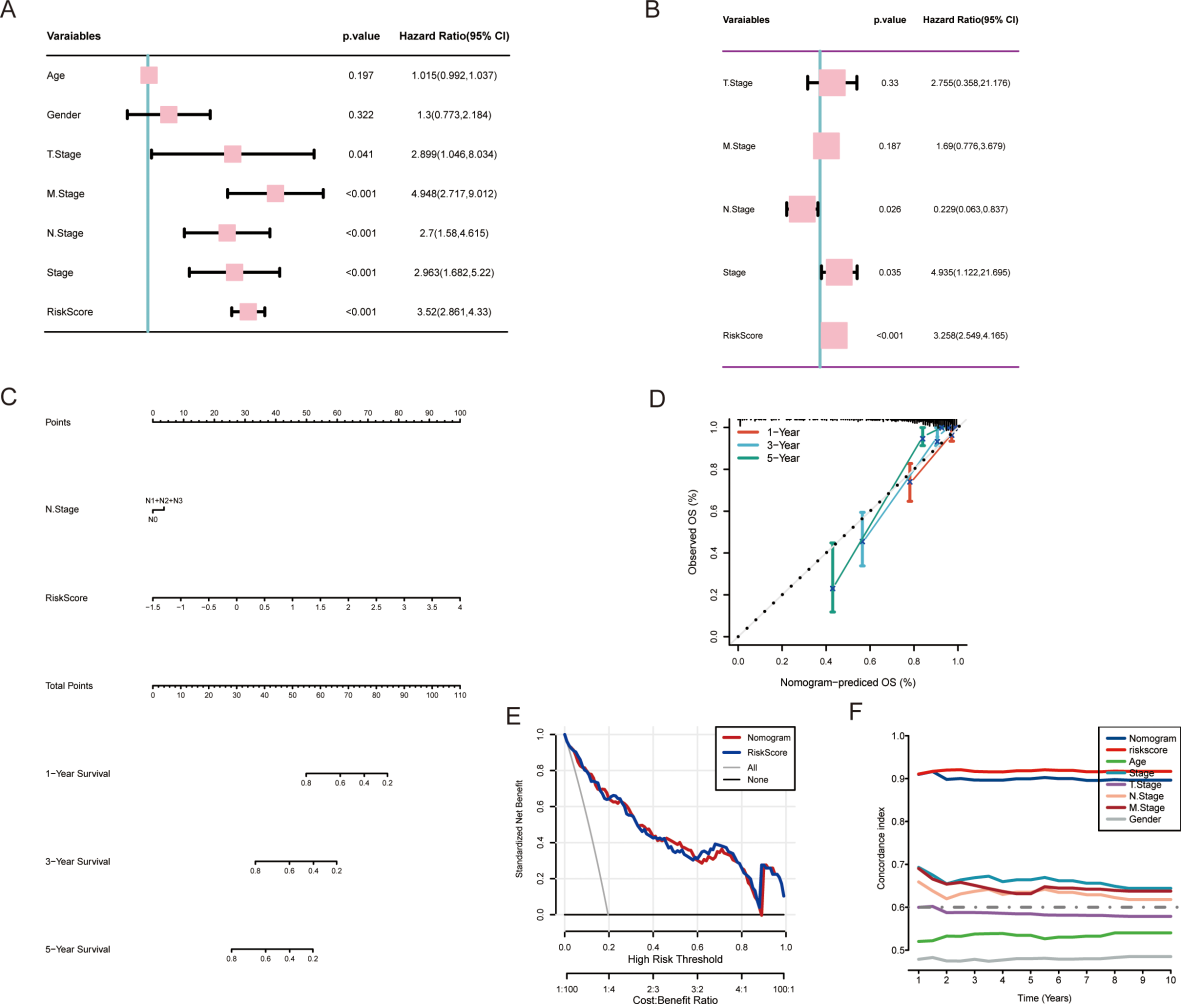
Establishment and verification of the nomogram. **(A, B)** Analysis of OS through univariate and multivariate evaluations of clinical features and the MAAD prognostic model. **(C)** Development of a nomogram integrating the MAAD prognostic model with clinical features. **(D)** The calibration curve for predicting 1, 3, and 5-year overall survival (OS) using the nomogram. **(E)** Decision curve analysis illustrates the net clinical benefit of utilizing the nomogram combined with the risk score. **(F)** Comparison of the nomogram and various clinical characteristics using the C-index.

To comprehensively evaluate the reliability of the MAAD score, we assessed its association with CRC patients by considering the MAAD score as an independent risk factor. We employed both univariate and multivariate regression analyses to evaluate the differences between the MAAD score and other clinical characteristics. The MAAD score was identified as an independent risk factor for OS in CRC patients (**Figures 5A, B**). To provide each CRC patient with a precise, digitized probability of survival or risk, we constructed a nomogram to assist clinicians in making individualized decisions (**Figure 5C**). The calibration curves illustrated a pronounced concordance between the predictive probabilities of our MAAD-related nomogram and the factual outcomes, which is evident in **Figure 5D**. Decision curve analysis (DCA) also indicated the reliable utility of the MAAD-related nomogram (**Figure 5E**). Furthermore, C-index analysis further confirmed the excellent performance of our nomogram model (**Figure 5F**).

### Analysis of potential pathway changes in MAAD

**Figure 6:**
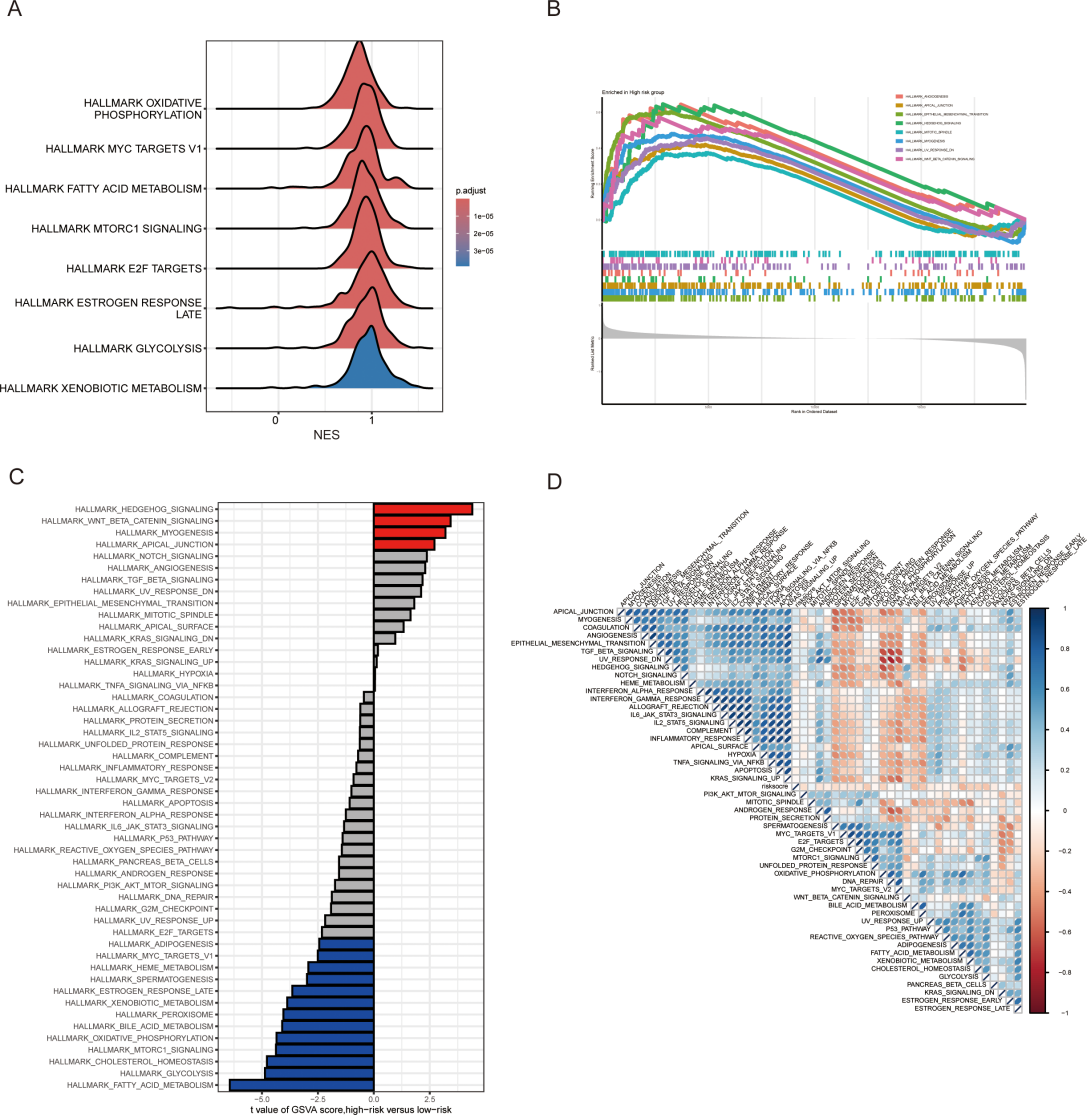
Potential pathway alterations under different MAAD prognostic models in colorectal cancer patients. **(A)** A ridge plot depicting the pathways predominantly enriched in the low-risk group. **(B)** Significantly enriched pathways in the high-risk group identified using GSEA. **(C)** Comparison of pathway activity differences between the high-risk and low-risk groups assessed by GSVA. **(D)** Relationship between the risk score and pathway activity levels analyzed through GSVA.

The molecular mechanisms of MAAD in CRC patients remain unclear. To provide a more comprehensive understanding of its impact on patients, we conducted a Gene Set Enrichment Analysis (GSEA) on patients across different risk groups. Interestingly, pathways such as oxidative phosphorylation, fatty acid metabolism, e2f targets, and glycolysis were significantly enriched in the low-risk group (**Figure 6A**). In contrast, angiogenesis, epithelial-mesenchymal transition, hedgehog signaling, and wnt/β-catenin signaling were significantly enriched in the high-risk group (**Figure 6B**). To further analyze the potential pathway activities, we used Gene Set Variation Analysis (GSVA). The results showed that hedgehog signaling and wnt/β-catenin signaling were significantly enriched in the high-risk group, while fatty acid metabolism, glycolysis, and oxidative phosphorylation were mainly enriched in the low-risk group (**Figure 6C**). The high consistency between GSEA and GSVA results further confirms the importance of these pathway activity changes in CRC patients. Subsequent correlation analysis between MAADs and pathway hallmark scores also further confirmed the close association of MAADs with metabolic pathways and tumor progression-related pathways in CRC (**Figure 6D**). These results may suggest that the differences in clinical characteristics between high and low MAAD risk groups may be related to these pathways.

### Intra-tumor heterogeneity and copy number variations analysis under MAAD mode

**Figure 7:**
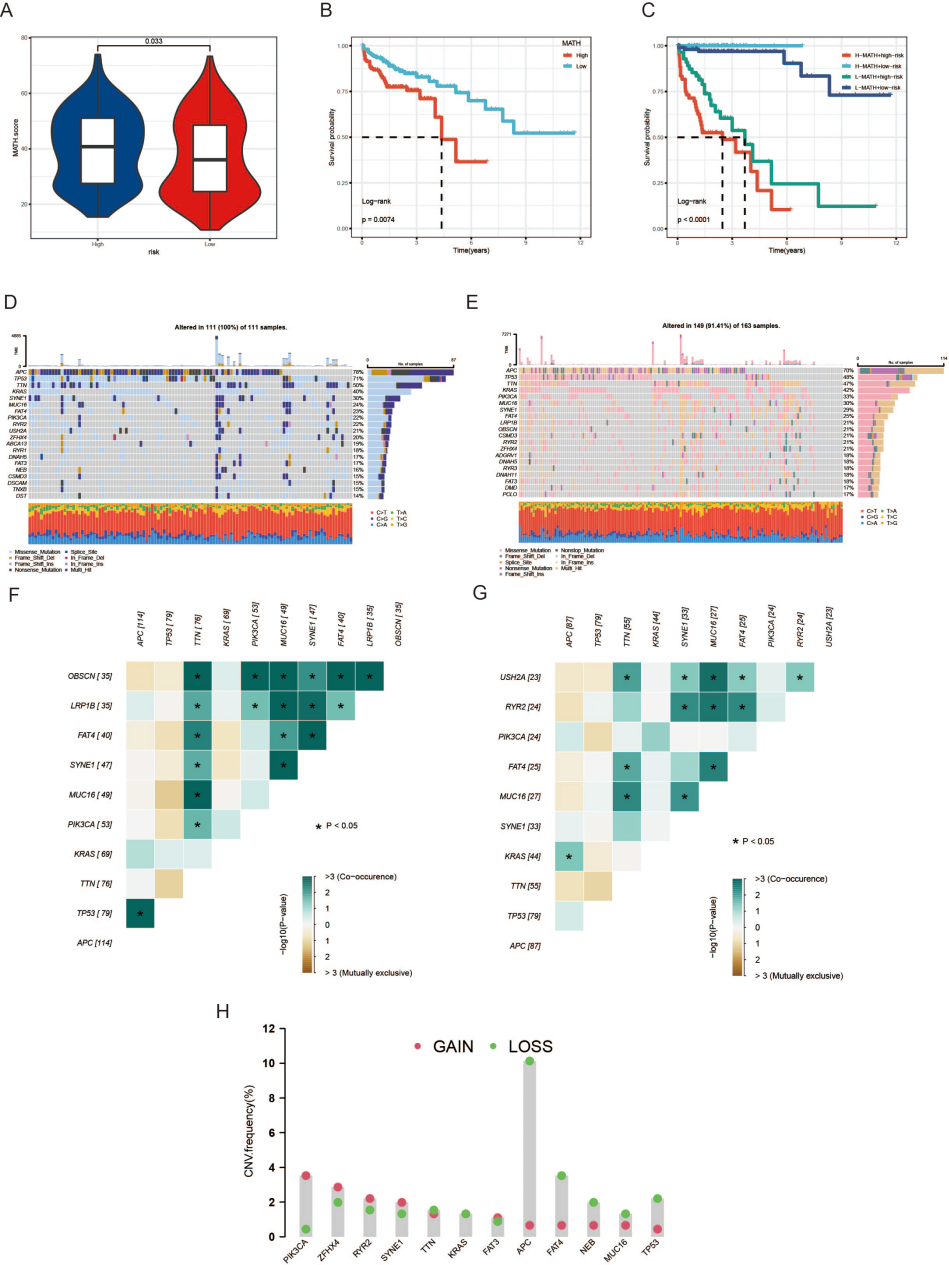
Analysis of tumor heterogeneity and genetic alterations associated with the MAAD prognostic model. **(A)** A violin plot showing the differences in MATH scores between the high-risk and low-risk groups. **(B)** OS differences between high- and low-risk groups. **(C)** OS analysis combining the risk score and MATH score. **(D, E)** Waterfall plots depicting the distribution of somatic mutations in colorectal cancer patients, with **(D)** representing the high-risk group and **(E)** the low-risk group. **(F, G)** Heatmaps showing the distribution of CNV frequencies for the top 12 genes in the high-risk group **(F)** and the low-risk group **(G)**. **(H)** CNV frequency of 12 genes. *p < 0.05.

CRC often exhibits intra-tumor heterogeneity (ITH), which typically arises from the accumulation of genetic mutations. This heterogeneity affects tumor occurrence, progression, metastasis, and response to treatment on multiple levels. To comprehensively elucidate the role of ITH in high and low-risk groups, we employed the MATH algorithm for scoring. The high-risk group exhibited notably elevated MATH scores in comparison to the low-risk group, as depicted in **Figure 7A**. In addition, CRC patients with increased MATH scores had a markedly lower survival rate than their counterparts with decreased scores, a comparison detailed in **Figure 7B**. Subsequently, we integrated ITH and MAAD scores to enhance the predictive accuracy of CRC and provide more precise diagnoses for patients. Patients in the low-risk + low MATH group had significantly better prognostic survival rates than those in the high-risk + high MATH group (**Figure 7C**). We then analyzed the mutational landscape between the high and low-risk groups. TP53, the most common tumor suppressor gene, had a mutation frequency of 71% in the high-risk group, significantly higher than the 48% in the low-risk group (**Figures 7D, E**). Additionally, the frequency of co-occurrence mutations was higher in the high-risk group than in the low-risk group (**Figures 7F, G**). The 12 genes with the greatest differences between the high and low-risk groups were selected for CNV analysis. **Figure 7H** shows that PIK3CA, ZFHX4, RYR2, and SYNE1 had higher CNV gains than CNV losses, while APC, FAT4, NEB, MUC16, and TP53 had higher CNV losses than CNV gains (**Figure 7H**).

### The relationship between MAAD and the tumor microenvironment and immunotherapy response

**Figure 8:**
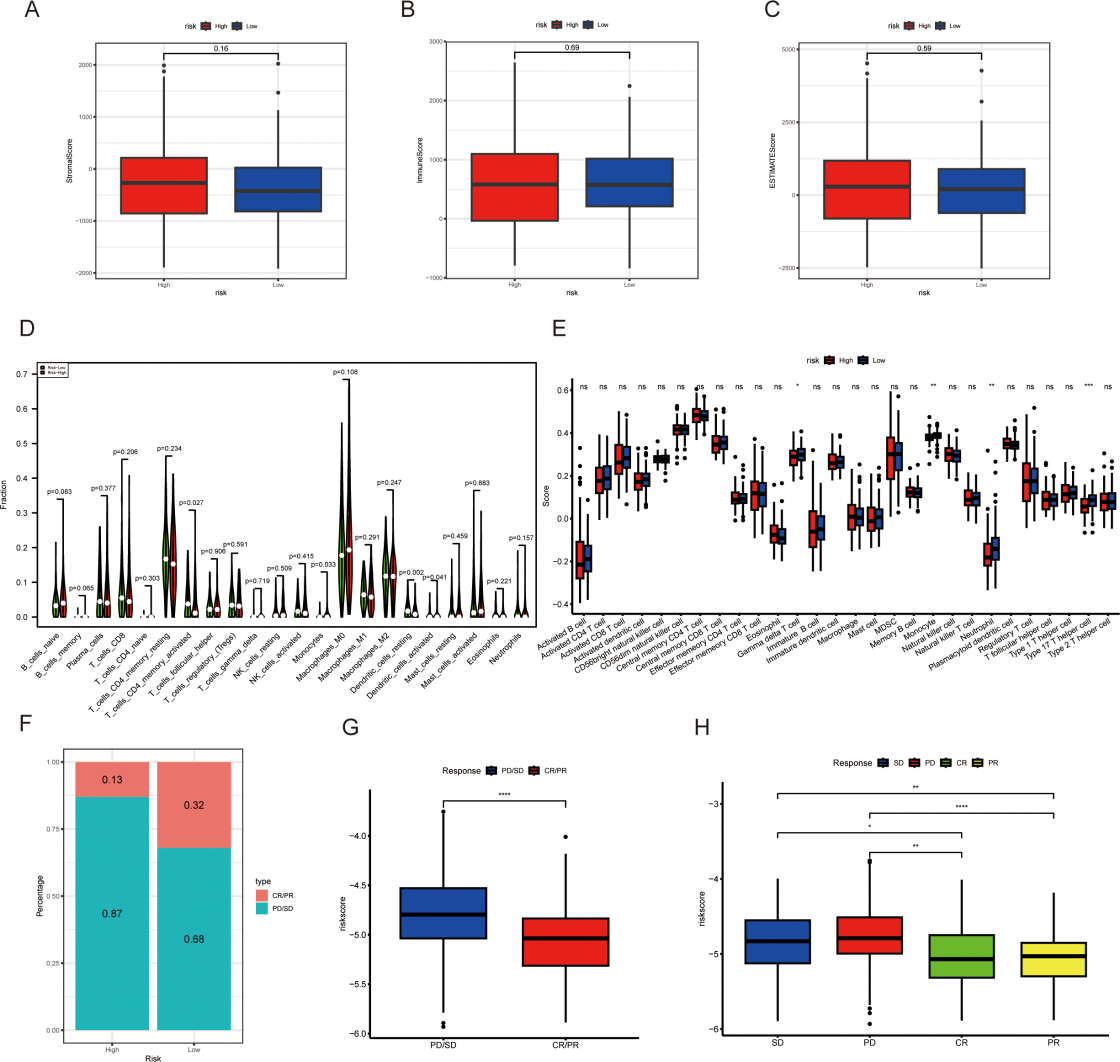
The relationship between the MAAD prognostic model and immune microenvironment and immunotherapy response. **(A)** Stromal scores in high- and low-risk groups. **(B)** Immune scores in high- and low-risk groups. **(C)** Estimate scores in high- and low-risk groups. **(D, E)** Immune cell infiltration in high- and low-risk groups of colorectal cancer patients, based on the CIBERSORT algorithm **(D)** and the ssGSEA algorithm **(E)**. **(F)** Proportion of CR/PR and PD/SD patients receiving immunotherapy in the high- and low-risk groups from the IMvigor210 cohort. **(G)** A boxplot showing the difference in risk scores between CR/PR patients and PD/SD patients in the IMvigor210 cohort. **(H)** Risk scores among CR, PR, SD, and PD patients based on the IMvigor210 cohort. *p < 0.05, **p < 0.01, ***p < 0.001, ****p < 0.001, with ns indicating no statistical significance.

Dynamic changes in the immune microenvironment during the progression of CRC are crucial for the response to immunotherapy. Therefore, we scored patients in high and low-risk groups for stromal score, immune score, and estimate score. The results showed no significant differences in these three scores between the high and low-risk groups (**Figures 8A-C**). Subsequently, we analyzed the immune cell infiltration in the high and low-risk groups. The CIBERSORT results showed that the proportions of memory activated CD4 T cells and resting dendritic cells were significantly higher in the low-risk group than in the high-risk group (**Figures 8D**). The ssGSEA results indicated that the score for gamma delta T cells was significantly higher in the low-risk group than in the high-risk group (**Figures 8E**). We evaluated the ability of the MAAD model to predict the response to immunotherapy by analyzing the IMvigor210 cohort treated with atezolizumab. Based on the MAAD model, we divided the patients in the IMvigor210 cohort into high and low-risk groups. It emerged from our data that the low-risk group had a reduced frequency of progressive disease and stable disease (PD/SD). By contrast, there was a notable surge in the group’s complete and partial response (CR/PR) figures, which is highlighted in **Figure 8F**. Individuals categorized as PD/SD tended to have lower risk scores than those classified as CR/PR (**Figures 8G, H**).

### Cellular validation experiments

**Figure 9:**
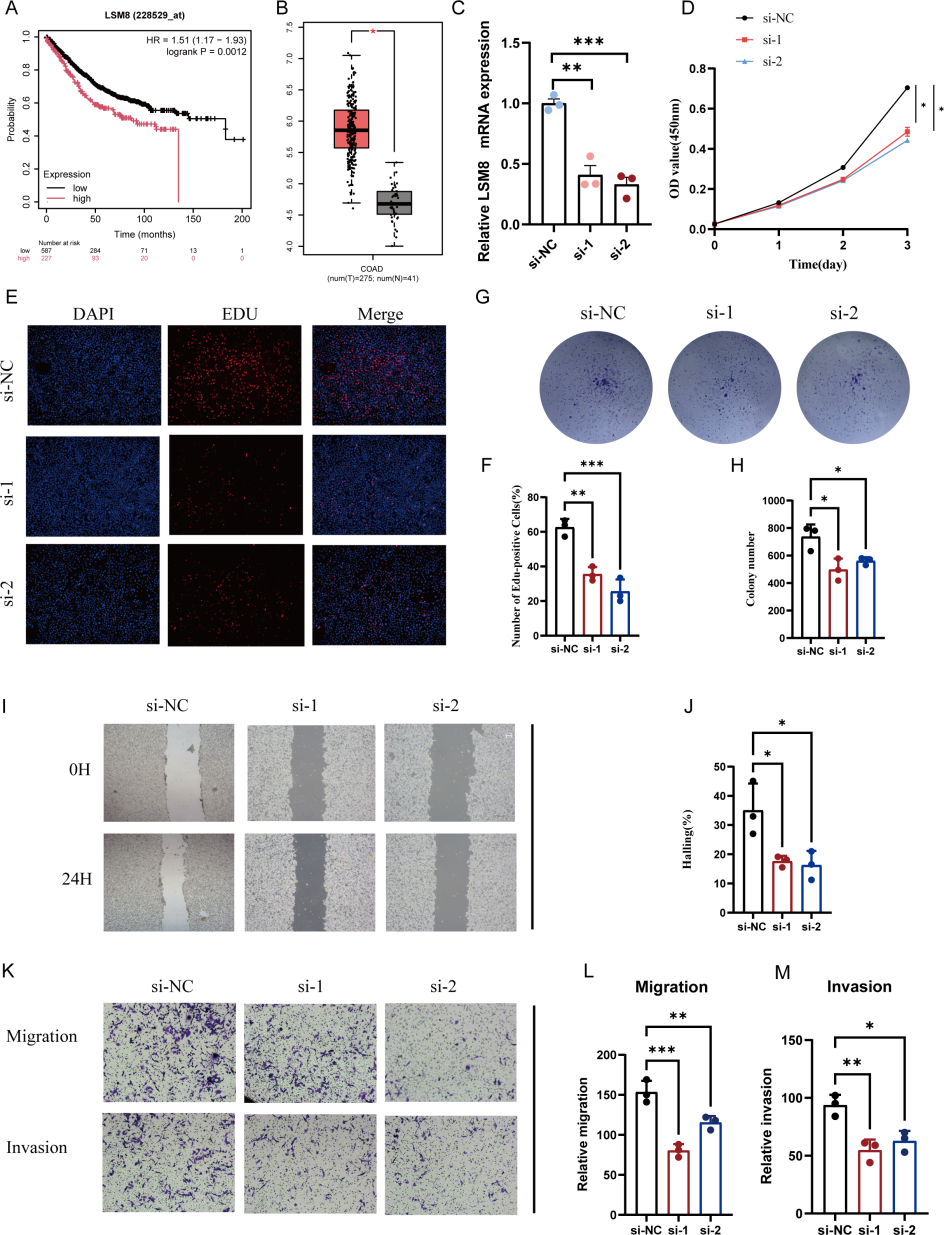
Reduction of LSM8 expression suppresses the malignancy of CRC cells. **(A)** Survival analysis showing the effect of LSM8 on the OS of CRC patients. **(B)** LSM8 expression comparison in normal versus tumor samples. **(C)** qRT-PCR to assess LSM8 knockdown efficiency, showing siRNA significantly reducing LSM8 expression. **(D)** Knockdown of LSM8 expression decreases RKO cell line viability as indicated by CCK8 assays. **(E, F)** EdU staining reveals that reducing LSM8 expression diminishes RKO cell line proliferation. **(G, H)** Representative images of colony formation assays in RKO cells transfected with shNC, si-1, and si-2. **(I, J)** Representative images and quantification of wound healing assays in RKO cells at 0 hours (0H) and 24 hours (24H) post-scratch. **(K-M)** Representative images and quantification of transwell migration and invasion assays in HT-29 and RKO cells. Cells transfected with siNC, si-1, and si-2 were analyzed. *P < 0.05, **P < 0.01, ***P < 0.001.

Following further screening of the genes in the prognostic model, we identified that high expression of LSM8 correlates with poorer patient survival prognosis, and LSM8 is also highly expressed in tumor tissues (**Figures 9A, B**; **Supplementary Figure S1**). Consequently, we performed LSM8 knockdown in the RKO cell line (**Figure 9C**). CCK-8 and EdU assays indicated that LSM8 knockdown reduces the proliferation rates of tumor cells (**Figures 9D-F**). Additionally, LSM8 knockdown diminished the colony-forming capacity of the RKO cell line (**Figures 9G, H**). We also assessed the malignant indicators of tumor cells—migration and invasion capabilities. The results showed that LSM8 knockdown significantly inhibits the migration and invasion abilities of tumor cells (**Figures 9I-M**).

## Discussion

CRC poses a significant challenge in the global health sector, dealing a heavy blow to human life and bringing about considerable economic and social burdens. Due to the lack of significant clinical symptoms in the early stages of CRC, many patients are diagnosed at an advanced stage, which greatly increases the difficulty of treatment (17, 18). The disease typically originates from the glandular epithelium of the colon or rectum and gradually progresses to an invasive tumor through the adenoma-carcinoma sequence, exhibiting significant heterogeneity. As the tumor progresses, common occurrences include invasion of the bowel wall layers and metastasis to distant organs (19). The heterogeneity and complexity of the tumor make traditional treatment methods limited in effectiveness, especially in late-stage patients, where treatment faces significant challenges (17, 18). Therefore, gaining a deeper understanding of the mechanisms underlying the development of cancer and identifying new therapeutic targets has become an important direction in current medical research (20, 21). In recent years, the metabolism of MAAD has been found to be closely related to the occurrence and progression of CRC. MAAD not only participates in the energy supply, protein synthesis, and biosynthesis of tumor cells but also plays a key role in regulating the proliferation, migration, metastasis, and immune escape of tumor cells (22, 23). Through in-depth research on the relationship between MAAD and CRC, this study aims to provide new theoretical evidence for the early diagnosis, precision treatment, and targeted intervention of CRC, and to offer more ideas and potential targets for clinical treatment.

The rapid development of high-throughput sequencing has provided more new insights and therapeutic strategies in the field of medicine (24, 25). Recently, the application of machine learning in the medical field has become increasingly mature (26, 27). Machine learning can effectively integrate complex information in high-throughput sequencing data and mine gene features closely related to prognosis (28, 29). The heterogeneity of tumors often leads to significant differences in treatment outcomes for patients. This heterogeneity exists not only between tumors of different patients but also among different cells within the same tumor. With the continuous development of single-cell sequencing technology, it is now possible to reveal the degree of variation and interconnections between different cells within a tumor, as well as changes in cell developmental trajectories at the single-cell level (30, 31). The variability of metabolic pathways within tumors has a profound impact on the occurrence, development, and treatment of cancer. With the continuous updating and iteration of bioinformatics algorithms, it has become easier to accurately observe changes in specific metabolic pathways. The AUCell algorithm can calculate the activity score of specific metabolic pathways for each cell, helping researchers identify new metabolic biomarkers and potential therapeutic targets (32). Previous studies have explored the impact of lactylation-related signaling changes on the prognosis and immune microenvironment of colorectal cancer patients (33).

Therefore, based on the analysis of single - cell sequencing results from CRC patients and using the AUCell algorithm, we comprehensively assessed the activity of the MAAD pathway across different immune cells. This approach allowed us to observe the nuanced changes in the activity of the MAAD pathway within the tumor microenvironment. Subsequently, we employed the WGCNA algorithm to delve into the intricate association between the MAAD pathway and CRC. By focusing on molecules within key modules, we sought to uncover deeper biological significance and potential mechanisms underlying the observed associations. Furthermore, by integrating multiple machine learning algorithms, we significantly enhanced the predictive accuracy of the prognostic model. This integration enabled a more comprehensive understanding of the impact of MAAD changes on the progression of CRC. The enhanced model provided a more robust framework for understanding the complex mechanisms of the tumor. Notably, patients in the low - risk group exhibited longer survival, better response to immunotherapy, and lower tumor heterogeneity scores. These findings further highlight the potential significance of MAAD activity changes in the context of CRC and underscore the importance of our research in revealing the complex mechanisms of the disease. In the high-risk group, the angiogenesis, epithelial-mesenchymal transition, hedgehog signaling, and wnt/β catenin signaling pathways were significantly enriched. Tumor cells promote angiogenesis by secreting dickkopf2 to support tumor cell growth (34). Epithelial-mesenchymal transition is a key biological process that plays an important role in tumor occurrence and development, mediating chemotherapy resistance through RHOJ-regulated epithelial-mesenchymal transition (35). Berberine can inhibit the hedgehog signaling pathway to weaken the malignancy of cells (36). SLC26A9 can affect the growth cycle of colorectal cancer cells by regulating the wnt/β catenin signaling pathway (37). Cellular experimental results showed that the proliferation, migration, and invasion capabilities of RKO cells were significantly reduced after LSM8 knockdown. In gastric cancer patients, high levels of LSM8 expression were associated with fewer immune cell infiltrations (38). This may suggest that elevated LSM8 expression may play a role in suppressing the immune response within the tumor microenvironment.

This study integrates single-cell transcriptomics with bulk transcriptomics data, focusing on the fluctuations in MAAD activity, and employs diverse machine learning algorithms to construct a predictive model. Through cellular experiments, we have gained insights into the potential functions of LSM8 in colorectal cancer cell lines, thereby expanding the potential clinical treatment strategies for colorectal cancer. Despite these findings, our study has its limitations. At present, our research is concentrated on the regulatory effects of LSM8 on the phenotypes of colorectal cancer cell lines, but the analysis of its downstream signaling pathways is not yet sufficiently in-depth. In the future, we will commit to exploring this area more thoroughly in order to fill the gaps in the current research.

## Conclusion

This study combined multi-omics analysis and machine learning algorithms to analyze the relationship between MAAD activity and colorectal cancer. We constructed a prognostic model based on MAAD-related genes and comprehensively analyzed tumor heterogeneity, immune microenvironment, immune therapy response, and potential pathway changes under different risks. Finally, cell experiments confirmed that knocking down the expression of the MAAD-related gene LSM8 can reduce the malignancy of CRC cell lines.

## Data Availability

The datasets presented in this study can be found in online repositories. The names of the repository/repositories and accession number(s) can be found in the article/**Supplementary Material**.

## Supplementary material

Supplementary Figure 1 Prognostic survival analysis of key genes.
