# Supplementary figures and images for: Integrating multi-omics and machine learning methods reveals the metabolism of amino acids and derivatives-related signature in colorectal cancer
Source: Front Oncol. 2025 Mar 26;15:1565090. doi: 10.3389/fonc.2025.1565090 (PMC11978647; doi:10.3389/fonc.2025.1565090)

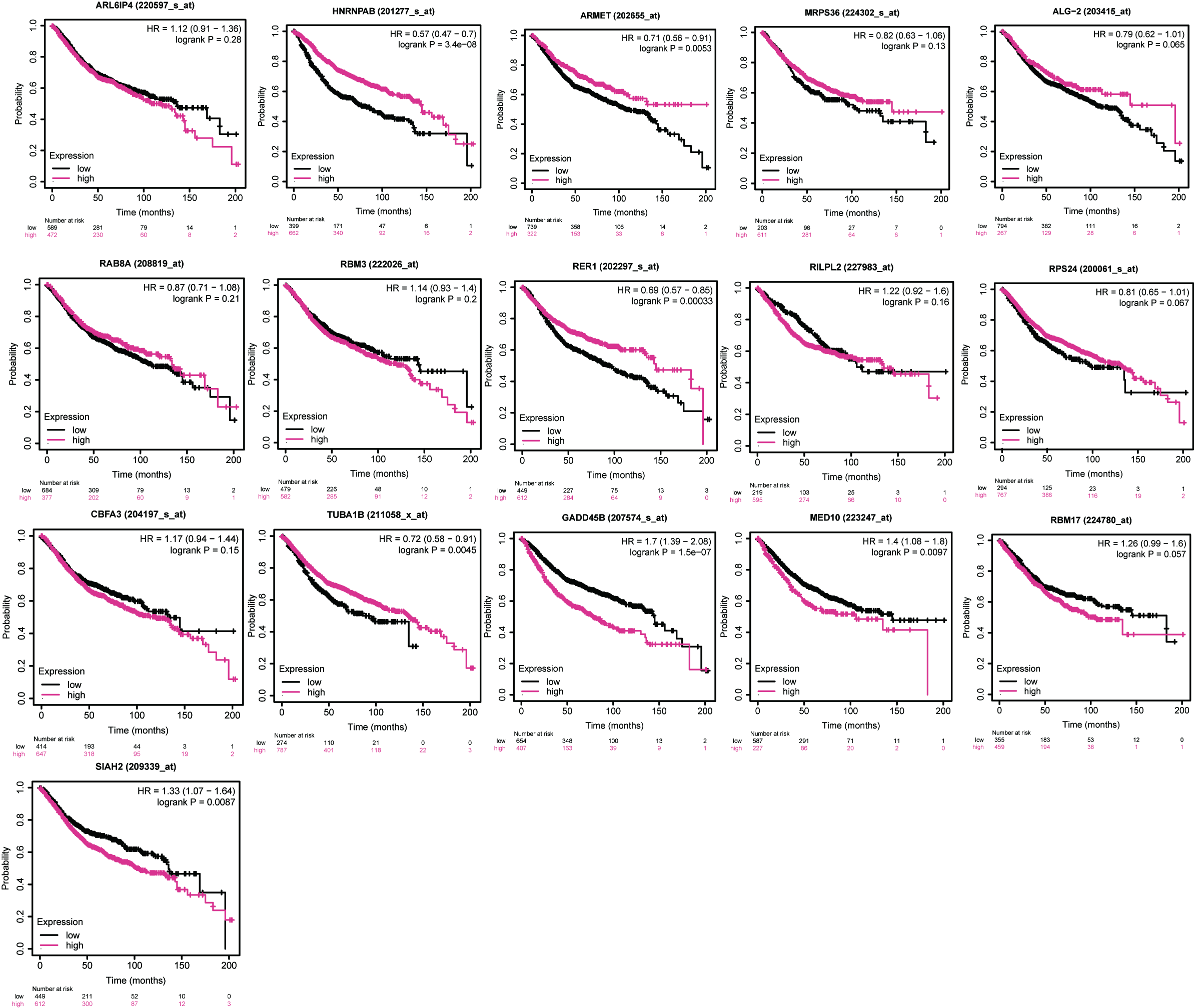

Supplement: Supplementary Figure 1 — Prognostic survival analysis of key genes. [file Image1.tif]
